# Efficacy and cost-effectiveness of a web-based and mobile stress-management intervention for employees: design of a randomized controlled trial

**DOI:** 10.1186/1471-2458-13-655

**Published:** 2013-07-15

**Authors:** Elena Heber, David Daniel Ebert, Dirk Lehr, Stephanie Nobis, Matthias Berking, Heleen Riper

**Affiliations:** 1 Division of Online Health Training, Innovation Incubator, Leuphana University, Lueneburg, Germany; 2 Department of Clinical Psychology and Psychotherapy, Philipps-University, Marburg, Germany; 3 Department of Clinical Psychology, VU University, Amsterdam, The Netherlands; 4 Institute for Health and Care Research (EMGO), VU University Medical Centre, Amsterdam, The Netherlands

**Keywords:** Internet intervention, Efficacy, Prevention, Work-related stress, Stress management, Depression, Occupational health, Cost-effectiveness

## Abstract

**Background:**

Work-related stress is associated with a variety of mental and emotional problems and can lead to substantial economic costs due to lost productivity, absenteeism or the inability to work. There is a considerable amount of evidence on the effectiveness of traditional face-to-face stress-management interventions for employees; however, they are often costly, time-consuming, and characterized by a high access threshold. Web-based interventions may overcome some of these problems yet the evidence in this field is scarce. This paper describes the protocol for a study that will examine the efficacy and cost-effectiveness of a web-based guided stress-management training which is based on problem solving and emotion regulation and aimed at reducing stress in adult employees.

**Methods:**

The study will target stressed employees aged 18 and older. A randomized controlled trial (RCT) design will be applied. Based on a power calculation of d=.35 (1-β of 80%, α = .05), 264 participants will be recruited and randomly assigned to either the intervention group or a six-month waitlist control group. Inclusion criteria include an elevated stress level (Cohen’s Perceived Stress Scale-10 ≥ 22) and current employment. Exclusion criteria include risk of suicide or previously diagnosed psychosis or dissociative symptoms. The primary outcome will be perceived stress, and secondary outcomes include depression and anxiety. Data will be collected at baseline and seven weeks and six months after randomization. An extended follow up at 12 months is planned for the intervention group. Moreover, a cost-effectiveness analysis will be conducted from a societal perspective and will include both direct and indirect health care costs. Data will be analyzed on an intention-to-treat basis and per protocol.

**Discussion:**

The substantial negative consequences of work-related stress emphasize the necessity for effective stress-management trainings. If the proposed internet intervention proves to be (cost-) effective, a preventative, economical stress-management tool will be conceivable. The strengths and limitations of the present study are discussed.

**Trial registration:**

German Register of Clinical Studies (DRKS): DRKS00004749

## Background

Stress at work is known to almost every employee. Shorter periods of work-related stress elicit acute stress reactions but are not hazardous to health and can even enhance the workers’ ability to meet challenges at work. However, if stress passes into a chronic state of tension, serious negative health consequences can result [1-3].

The prevalence rate of stress in employees varies considerably depending on several factors, such as the definition of stress, the measurement that is used or the occupation of the workers. A European survey of 21,703 workers showed that 28% of workers experience work-related stress, and the percentages range from 17% in elementary occupations to 40% in professionals [4].

A meta-analysis found that job strain and effort-reward imbalances produce consistent increases in the risks for common mental disorders [3]. Work stress may precipitate diagnosable depression and anxiety in workers who were previously healthy [5]. Furthermore, sleep disturbances are a common negative consequence of stress [6]. Moreover, work-related stress can have adverse effects on a societal level due to sickness leave [7] and work disability [8]. According to the American Institute of Stress, the cost estimates of job stress amount to “over $300 billion annually due to increased absenteeism, employee turnover, diminished productivity, medical, legal, and insurance expenses, and workers' compensation payments.” [9]

A variety of effective traditional occupational stress-management trainings are available [10,11]. An extensive meta-analysis [10] showed that the effectiveness of these trainings varies depending on the type of intervention and outcome measure applied. The efficacy of these interventions ranges from d=.11 (the effect of relaxation on depression) to d=.70 (the effect of cognitive behavioral therapy on anxiety) and the overall effect size across all studies is d=.34. However, occupational stress-management interventions are often taught to only a small group of people who are guided by an instructor and these interventions take place at a specific location [11].

Compared to face-to-face interventions, web-based guided self-help interventions offer the advantages of being independent of time, location and group. Furthermore, web-based guided self-help interventions have shown moderate to large effect sizes in numerous studies of, for example, depression and anxiety [12]. A number of randomized controlled trials on web-based stress-management interventions have been performed, including studies on general populations of people with stress-related complaints [13-15] or work-related stress [16], college students [17], women [18], families [19,20], and individuals with burnout symptoms [21]. Thereby, the effect sizes for studies that use stress as an outcome measure vary from non-significant [17] to moderate (d = .60) [16]. However, only few studies have investigated the potential of web-based stress-management interventions in employees [22-27]. These studies of employees apply a wide range of outcome measures, including stress, depression, anxiety, and physiological measurements. With regard to the respective outcome measure, the results again vary between non-significant for stress [23,26] to moderate for depression [24].

To date, the cost-effectiveness of worksite mental health interventions has been studied only in a limited number of trials [28]. A recent systematic review [28] found that these interventions might be cost-effective; however, more high-quality economic evaluations are needed to draw firm conclusions. Data on cost-effectiveness has not been presented in any of the afore-mentioned studies on web-based stress-management for employees. For web-based interventions, studies currently investigate the cost-effectiveness of mental health interventions for workers, including studies of a workers’ health surveillance mental module [29] and a guided self-help course for workers with depressive symptoms [30]. However, to the best of our knowledge, there are no studies with full economic evaluations of the cost-effectiveness of web-based stress-management training for employees.

The theoretical basis of web-based interventions for stress is diverse and ranges from cognitive behavioral methods [16,22,24,27], mindfulness [15,31], problem solving [13], social cognitive theory [23,32] to health behavior change theory [23]. Surprisingly however, there are currently no web-based interventions with content based on specific stress models, such as the job-demand control model [33], the effort-reward imbalance model [34] or more generic models of stress, such as the transactional model of stress [35]. Lazarus’ transactional model of stress [35] can be applied to working contexts and other life areas. This model identifies two strategies of coping with stressors: problem- and emotion-oriented coping. Some web-based stress-management interventions have already included problem solving as part of their intervention content [14,24,32,36] or as stand-alone interventions [13]. However, web-based stress-management interventions employing a combination of problem- and emotion-oriented coping methods currently do not exist.

At work, people often must not only cope with difficult situations and solve problems but also face challenging emotions that arise from these situations. Deficits in emotion regulation skills are an important factor in the development and maintenance of a broad range of mental disorders, such as depression [37-39] and anxiety [40]. As deficits in the ability to adaptively cope with difficult emotions are related to various mental health problems [41], the usage of emotion regulation techniques in a low-threshold web-based stress-management training to reduce symptoms of distress appears promising.

The available web-based stress-management interventions cover a wide range of primary treatment components, including several cognitive behavioral methods [16,21,22,24]. In addition, some interventions focus not only on stress but also on health behavior problems such as alcohol abuse [22,23] and unhealthy diets [23]. A meta-analysis [11] found that occupational stress-management interventions that focus on fewer treatment components are more effective than interventions that include several components. The authors of this meta-analysis found combined effect sizes of d=.64 and d=.61 for the effectiveness of interventions with one or two treatment components, respectively; and treatments with four or more components yielded a smaller effect size of d=.27 [11]. Thus, it appears promising to opt for fewer intervention components in stress-management interventions.

### Aims of the study and hypotheses

In this study, we will investigate the efficacy and cost-effectiveness of a newly developed web-based stress-management training for stressed employees that is based on problem solving and emotion regulation. As a theoretical basis we have followed the transactional stress model of Richard Lazarus [35]. We hypothesize that participants in the intervention group will show a greater reduction in the primary outcome perceived stress from pre-test to post-test compared to a waitlist control group, and that this effect will be maintained over six months. We will conduct cost-effectiveness and cost-utility analyses from a societal perspective and hypothesize that the intervention will be cost-effective. Furthermore, explorative moderator analyses will be conducted.

## Methods

### Study design

**Figure 1:**
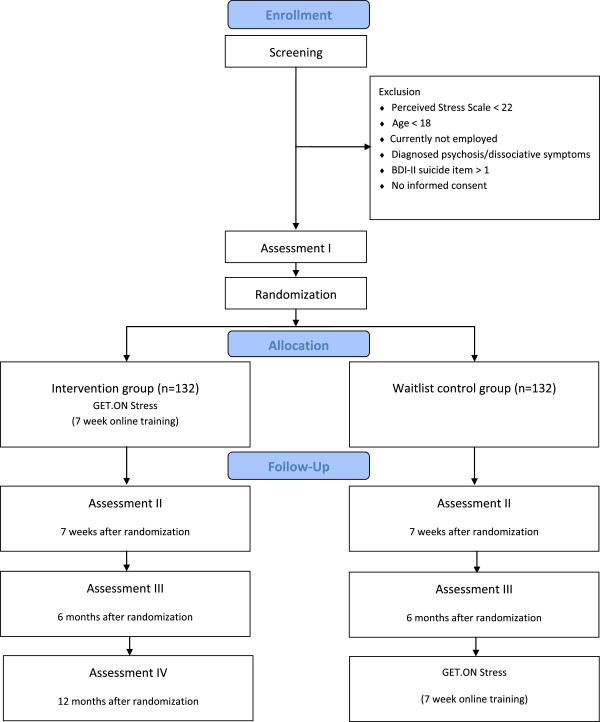
Study flow chart.

A randomized controlled trial with two conditions will be conducted. The intervention group will receive the web-based stress-management training “GET.ON Stress” and the control group will obtain access to this intervention after 6 months (see Figure 1). There will be no restriction with regard to the use of medication or other treatment as usual (TAU) (e.g., psychotherapy) in either of the groups. To control for potential confounding effects, TAU will be monitored.

### Inclusion and exclusion criteria

The target group will consist of stressed employees from the general working population aged 18 years or older. Inclusion criteria will include current employment and a distinct level of perceived stress as indicated by scores of 22 and above on the Perceived Stress Scale (PSS-10). This cut-off was chosen to select participants with a marked level of subjective stress as identified by one standard deviation (SD = 6.2) above the mean (PSS-10 = 15.3) in a large working population [42]. Applicants with an elevated risk of suicide (Beck Suicide Item > 1) and applicants that self-report having been diagnosed with psychosis or dissociative symptoms in the past will be excluded. For applicants that are at risk for suicide we follow a standardized procedure. These applicants will be advised to seek help via their general practitioner, the local psychiatric emergency room or an official emergency number, and the relevant information and telephone numbers will be provided by email.

### Procedure

Participants will be recruited from the general working population via mass media (e.g., newspaper articles, television) between March and October 2013. Moreover, a major health insurance company (BARMER GEK) will support the recruitment process within their occupational health management program. On an open access website (http://www.geton-training.de) the interested people will be able to sign up to participate with an email-address. Applicants will receive further informational material concerning the study conditions and the training and will be asked to provide an email-address and a first and last name (which can be pseudonyms if desired) to participate. Afterwards, a link to the screening questionnaire will be sent. Applicants who are eligible for participation will be required to provide their informed consent and fill out the online baseline questionnaire. Subsequently, these participants will be randomly allocated to either the intervention or the control group. Participants will be informed of the outcome of the randomization and participants in the intervention group will receive immediate access to the GET.ON Stress training. Assessments will be scheduled at seven weeks and six months after randomization for both groups. However, the participants in the control group will receive the login data required to complete the training six months later than the intervention group. An extended follow-up for the intervention group is scheduled 12 months post-randomization.

### Randomization

The randomization will be carried out by an independent researcher using a web-based randomization program (randomisation.eu) that will be set to allocate participants (N=264) into each group at a ratio of 1:1.

### Sample size

We aim to include 264 participants to produce a statistically relevant effect size of d = .35, a power (1-β) of 80% and an alpha of .05 (two-tailed test) for intention-to-treat analysis using PASS 12. A comprehensive meta-analysis on traditional face-to-face interventions for work-related stress yielded an overall combined effect size of d=.34, whereby the results for web-based stress-management interventions are mixed, ranging from non-significant [17] to moderate effect sizes [16]. Therefore, we aim for an effect size of d=.35.

### Intervention

The web-based GET.ON Stress intervention is based on two main components: problem solving and emotion regulation. The intervention consists of seven sessions composed of modules for psycho-education (session 1), problem solving (sessions 2–3), emotion regulation (sessions 4–6) and plan for the future (session 7) (see Table 1). Each session can be completed in approximately 45–60 minutes and consists of general information, examples related to work, interactive exercises, quizzes, audio and video files, and downloadable work sheets and mp3 files. We will recommend to log in once or twice a week and to fill out a short daily stress diary. Depending on the module, further homework will be assigned. Using responsive web-design, participants can follow the program on the internet, a tablet or mobile phone. An integrated read-aloud function allows participants to follow the lessons in an audio-narrated way.

**Table 1:** Content of the GET.ON stress training

**Session**	**Intervention content**	
1	Psycho-education	
2	Problem solving I	(Learning Phase)
3	Problem solving II	(Maintenance Phase)
4	Emotion regulation I	(Muscle- and breathing relaxation)
5	Emotion regulation II	(Acceptance and tolerance of emotions)
6	Emotion regulation III	(Effective self-support in difficult situations)
7	Plan for the future	

#### Psycho-education (session 1)

In the first session, the participants will be provided with psycho-educational information about stress based on Lazarus’ transactional model of stress [35], that includes emotion-focused and problem-focused coping strategies. Following a video introduction to the basic information about stress and coping methods, an interactive quiz will be presented to equip participants with general knowledge of the most appropriate coping strategies for common problem situations. The participants will identify their personal stressors and define their goals and motivation during the training. Furthermore, participants will be asked to choose one positive activity each day.

#### Problem solving (session 2–3)

In sessions 2 and 3, the participants will work on their problem-solving skills. This module is based on problem solving therapy [43]. The participants will learn a systematic six-step problem-solving method that can be applied to their individual problems. This method has already been adopted in other web-based studies [13,44]. Typical scenarios involving work-related stress will be presented. The participants will fill out their own six-step procedures and use their personal solution in the time between the sessions. In session 3, the participants will have the opportunity to either work on the same problem as in the previous session or choose a new problem.

#### Emotion regulation (session 4–6)

In sessions 4 to 6, the participants will work on the emotion regulation module. The emotion regulation techniques are based on the Affect Regulation Training (ART) [37,45] and include muscle and breathing relaxation, acceptance and tolerance of emotions and effective self-support. ART has been shown to be effective as additional treatment component in cognitive-behavioral treatment for depression [37] and also as stand-alone intervention in at risk-populations (i.e., police officers) [46]. In each session, one strategy will be taught, and the participants will practice that strategy with a 15-minute audio file according to the instruction of an expert. The techniques will be introduced using examples of typical emotional reactions related to work contexts. Video files explaining the psychological background will be displayed. The participants will be asked to listen to the appropriate 15-minute audio file on a daily basis and will be provided with downloadable mp3 files.

#### Plan for the future (session 7)

In the last session, the participants will be asked to reassess their goals for the training. Furthermore, they will identify their personal warning signs for stress. Additionally, they will be asked to strengthen something important in their lives and write a letter to themselves about how they imagine their life will be after four weeks of applying the stress-management methods they have been trained in.

#### Optional booster session

Four weeks after completing the training, the participants will be given the option of completing a booster session and evaluating their training progress. They will be given the opportunities to evaluate the letter they wrote to themselves in the last training session, reassess their goals and make plans to continue to include positive activities in their daily lives.

#### Additional information

At the end of sessions 2 to 6, information and exercise sections about common stress-related topics will be provided to enhance the recognition value of the stress-management training. These sections are optional and will not be part of the main intervention. These sections will cover the following topics: time management, rumination and worrying, psychological detachment from work, sleep hygiene, rhythm and regularity of sleeping habits, nutrition and exercise, organization of breaks during work, and social support.

#### Text message coach

If desired, participants will receive automatic motivational text messages and small exercises on their mobile phones. These messages will support the participant in transferring the exercises of the training into their daily lives (e.g., short relaxation exercises: “Relax your muscles in your hands and arms for 3 seconds now. Follow your breathing and each time you breathe out, relax a little more“). The participants will have the opportunity to choose between “light coach” (one text message every other day) and “intensive coach” (2–3 text messages every day) options.

#### eCoach support

Within 48 hours, the participants will receive personalized written feedback on the exercises they have completed in each session from an eCoach. The eCoaches are psychologists and trained master’s-level psychology students and will follow guidelines about the feedback process that are defined according to the standardized manual for the intervention. The eCoaches will send reminders in cases in which the participants do not complete one session within 7 days. The training itself, and the communication between the participant and the eCoach will take place in a secured web-based platform (AES 256-bit encryption). The participants will have access to the platform based on their email-addresses and self-designated passwords.

### Primary and secondary outcomes

Primary outcome will be perceived stress. In secondary analyses, we will explore the effects of depression, anxiety, emotional exhaustion, emotion regulation, attitudes toward seeking professional psychological help, work engagement, psychological detachment, insomnia severity, worrying, and absenteeism/presenteeism. Economic analyses will be conducted assessing cost-effectiveness and cost-utility from a societal perspective including the costs of all types of health services and the costs that stem from productivity losses.

### Outcome measures

#### Perceived stress

The German version of the ten-item-Perceived Stress Scale (PSS-10) [47,48] will be used as a primary outcome measure. The PSS is “the most widely used instrument for measuring perceived stress” [47] and assesses the degree to which people perceive their lives as stressful, particularly regarding how “unpredictable, uncontrollable and overloading respondents find their lives” [49]. Cronbach’s alphas range for this scale from .78 to .91 [49]. The scale is based on Lazarus’ transactional model of stress and, therefore, fits well with the theoretical basis of the intervention. Participants in this study will be asked to answer questions relating to the past week as opposed to the past month to avoid confounding with the training period. Similar procedures have been adopted in previous studies [48].

#### Depression

Depressive symptoms will be measured with the German version of the Center for Epidemiological Studies’ Depression Scale (CES-D) [50,51]. This frequently used self-report instrument consists of 20 items that are answered on a four-point Likert scale referring to the previous week. Total scores range from 0 to 60. The internal consistency of this measure has been found to be α = .89 [51].

#### Anxiety

Anxiety symptoms will be measured with the German version of the anxiety subscale of the Hospital Anxiety and Depression Scales (HADS) [52,53]. This subscale contains seven items related to the previous week. Each item is scored from 0 to 3; thus, the scores range from 0 to 21. The HADS has been shown to have a high internal consistency of α = .80 [53].

#### Emotional exhaustion

The German version of the Maslach Burnout Inventory (MBI-GS-D) [54,55] will be used to measure emotional exhaustion, the basic stress dimension of burnout. This commonly used self-report instrument consists of five items and uses a six-point Likert-type scale anchored by 1 = “never” and 6 = “very often”. The internal consistency of this subscale was α = .85 in a German sample [56].

#### Emotion regulation

To assess emotion regulation skills we will use the German Emotion Regulation Skills Questionnaire (ERSQ-27 and ERSQ-ES) [57,58]. The subscales of acceptance, emotional self-support and comprehension will be included to assess the usage of general emotion regulation skills during the previous week and measured on a five-point Likert-type scale (ranging from “not at all” to “almost always”). Each scale is comprised of three items. The overall Cronbach’s α of the ERSQ-27 was .90 in a community-based sample, and for the subscales of acceptance, emotional self-support and comprehension .68, .72 and .73, respectively [57]. Furthermore, the subscale “general distress” from the emotion-specific version of the ERSQ (ERSQ-ES) [58] will be used to assess the regulation of stress. Therefore, the general distress subscale containing 12 items will be applied to assess constructive coping with stress and tension within the last week on a five-point Likert-type scale (ranging from “not at all” to “always”). This subscale has a high internal consistency of α = .85 [58].

#### Attitudes toward seeking professional psychological help

We will measure the influence of attitudes toward mental health care service utilization with the ten-item Attitudes Toward Seeking Professional Psychological Help Scale – Short Version (ATSPPHS-SF) [59]. The items of this scale are answered on a four-point Likert scale and result in total scores of 0–30; higher scores indicate more positive attitudes. In a normative sample, this scale showed an adequate internal consistency (α = .84).

#### Work engagement

The Utrecht Work Engagement Scale (UWES) [60] assesses work engagement defined as a “positive, fulfilling, work-related state of mind that is characterized by vigor, dedication, and absorption” [61]. This scale has nine items, and the internal consistency of the total score is α = .91.

#### Psychological detachment

Psychological detachment from work will be measured with a subscale of the Recovery Experience Questionnaire (REQ) [62]. This subscale contains four items that are rated on a five-point Likert scale. The internal consistency of this subcscale is α = .85 [62].

#### Insomnia severity

Insomnia severity will be measured with the Insomnia Severity Index (ISI) [63,64]. This seven-item scale measures the nature, severity and impact of insomnia and is rated on a five-point Likert scale. The total score indicates overall insomnia severity and ranges from 0 to 28. This scale has been validated as a web-based measure [65]. Internal consistency has been found to be Chronbach’s α =.90 and .91 [66].

#### Worrying

We will use the ultra-brief three-item version of the Penn State Worry Questionnaire (PSWQ) [67,68] to assess worrying. Each item is answered on a seven-point scale. We have adapted the questionnaire to encompass the previous week based on the three items of the Penn State Worry Questionnaire-Past Week (PSWQ-PW) [69,70]. Total scores range from 0-18. The psychometric properties of the English ultra-brief version are similar to the standard English 16-item version (Cronbach’s α = .85 compared to α = .91 for the standard version) [68] and the German version of the PSWQ-PW.

#### Quality of life

We will use the Short Form 12 (SF-12) [71] and the EuroQol (EQ-5D) [72] to assess quality of life. The SF-12 [71] covers eight health domains (physical functioning, role limitations, pain, general health perception, vitality, mental health, emotional role and social functioning) and allows for the calculation of two sum scores for physical and mental health. We will also use the EQ-5D which is a widely applied, valid and reliable measurement of quality of life and consists of five items related to mobility, self-care, common activities, pain/discomfort and anxiety/depression. Furthermore, this measurement contains a visual analogue scale concerning health state.

#### Cost measures

The economic evaluation will be conducted from a societal perspective; thus, we will include direct medical (e.g., medicine), direct non-medical (e.g., parking) and indirect costs (e.g., productivity loss) [73] over the previous three months. We have adapted the Trimbos and Institute of Medical Technology Assessment Cost Questionnaire for Psychiatry (TiC-P) [74] for application to the German health care system.

#### Other measurements

Other measurements include demographic variables (e.g., age, gender, occupation etc.), the Effort Reward Imbalance Questionnaire – Short Form (ERI-SF) [75], the hope of improvement subscale of the German Patient Questionnaire on Therapy Expectation and Evaluation (PATHEV) [76] (adapted to the online training context), the German version of the Client Satisfactory Questionnaire (CSQ-8) [77,78] (adapted to the online training context), and a German questionnaire on the negative effects of psychotherapy (Ladwig, I., Rief, W., & Nestoriuc, Y.: Hat Psychotherapie auch Nebenwirkungen? Entwicklung des Inventars zur Erfassung Negativer Effekte von Psychotherapie (INEP) [Does psychotherapy have side effects? Development of an Inventory of Negative Effects of Psychotherapy (INEP)], submitted); adapted to the online training context). Approximately 30 minutes will be required to complete all questionnaires. For an overview of all outcome measures, see Table 2.

**Table 2:** Outcome measures

	**T0**	**T1**	**T2**	**T3**	**T4**
					
Perceived Stress Scale	✔	✔	✔	✔	✔
Center for Epidemiological Studies Depression Scale	-	✔	✔	✔	✔
Hospital Anxiety and Depression Scales – Anxiety	-	✔	✔	✔	✔
Maslach Burnout Inventory – Emotional Exhaustion	-	✔	✔	✔	✔
Emotion Regulation Skills Questionnaire – Comprehension/Acceptance/Self-Support	-	✔	✔	✔	✔
Emotion Regulation Skills Questionnaire – General Distress	-	✔	✔	✔	✔
Attitudes Toward Seeking Professional Psychological Help Scale	-	✔	✔	✔	✔
Utrecht Work Engagement Scale	-	✔	✔	✔	✔
Recovery Experience Questionnaire – Psychological Detachment	-	✔	✔	✔	✔
Insomnia Severity Index	-	✔	✔	✔	✔
Penn State Worry Questionnaire – Ultra Brief	-	✔	✔	✔	✔
Trimbos/iMTA Questionnaire for Costs associated with Psychiatric Illness	-	✔	-	✔	(✔)
Quality of Life (EuroQol, SF-12)	-	✔	-	✔	(✔)
Demographic Variables Questionnaire	✔	-	-	-	-
Effort Reward Imbalance Questionnaire – Short Form	-	✔	-	-	-
Patient Questionnaire on Therapy Expectation and Evaluation (Online-Training)	-	✔	-	-	-
Client Satisfactory Questionnaire	-	-	(✔)	-	-
Questionnaire on Negative Effects of Online Trainings	-	-	(✔)	(✔)	(✔)
Potential Dropout Reasons	-	-	(✔)	-	-

*T0* Screening, *T1* Baseline, *T2* 7 weeks, *T3* 6 months, *T4* 12 months.

Assessments: ✔ = intervention and control group, (✔) = intervention group only

### Statistical analyses

Analyses will be conducted and reported according to the Consolidated Standards of Reporting Trials (CONSORT) statement regarding eHealth [79,80].

#### Clinical analyses

Data will be analyzed on an intention-to-treat basis. Additionally, completers-only and per-protocol analyses will be carried out. Missing data will be dealt with following the recommendations of Little and Rubin [81] and Schafer [82]. We will use repeated measurements analysis of variance to examine differences in the primary and secondary outcome measures between the two groups, and we will compute standardized effect sizes (Cohen’s d). The number needed to treat (NNT) and the clinical significance in terms of a reliable change and recovery rates will be investigated using the method of Jacobson and Truax [83].

#### Moderator analyses

Moderator analyses will be conducted including potential moderators as interaction with treatment condition as independent variable in the main effect analyses. Potential moderators to be examined include severity of baseline stress symptoms, socio-demographic variables (e.g. age, gender, education etc.), sleep disturbances, emotion regulation skills, worrying and psychological detachment.

#### Economic evaluation

We will perform an economic evaluation from a societal perspective that includes all relevant costs and outcomes. A cost-effectiveness analysis and a cost-utility analysis will be conducted. For cost-utility analyses, quality-adjusted life years (QUALYs) will be calculated. A non-parametric boot-strapping method with 95% confidence intervals in percentiles will be used to assess differences between the intervention and control group. We will compare the intervention and control groups in terms of incremental costs and incremental effects. Towards this end, we will calculate the incremental cost-effectiveness ratio (ICER). We will use bootstrapping (5,000 times) to test the robustness of the ICER and to quantify the uncertainty in the ratios. The results will be shown in a cost-effectiveness plane and in a cost-effective acceptability curve. Additionally, the robustness of the base-case findings will be tested with a multi-way sensitivity analysis.

## Discussion

In this study, we will examine the efficacy and cost-effectiveness of a web-based stress-management training in highly stressed employees. We expect that the stress levels of participants in the intervention group will be significantly lower after the training and after six months compared to these measures from the control group. Furthermore, we expect this intervention to be cost-effective.

The intervention content of the stress training was developed with a clear theoretical background based on Lazarus’ transactional model of stress [35]. A recent study of a web-based intervention aimed at reducing stress provides psycho-educational information about problem- and emotion-focused coping in one out of 13 training sessions [15]. However, there are currently no web-based stress-management interventions that have used the combination of problem- and emotion-focused coping based on the definitions of Lazarus [35] as the main theoretical basis. In the research on stress, problem-oriented coping methods such as the problem-solving training have always been strongly emphasized. In addition, many interventions include cognitive restructuring as a treatment component. With this focus, emotions are only addressed indirectly as a sub-area of cognitions. Recently, deficits in emotion regulation skills as a treatment target have gained attention in the field of mental health [37,41]. Emotion regulation has been shown to be relevant in a broad range of mental disorders [41] including depression [37-39] and anxiety [40]. Only now, the targeting of emotions as an autonomous treatment component finds its way into stress-management training formats. A study in hypertensive employees found that a stress-management intervention consisting of positive emotion refocusing and emotional restructuring techniques can improve stress, depression, emotional health and systolic blood pressure [84]. Furthermore, an intervention designed to increase emotional competence has been found to result in decreased perceived stress and lower cortisol secretion in adulthood [85]. However, to the best of our knowledge, there is no web-based stress-management training available that has included emotion regulation as main treatment component. Thus, the web-based training proposed herein includes emotion regulation as an innovative and promising component in addition to the traditional, established component of problem solving.

The inclusion of an economic evaluation constitutes a strong advantage of this study. There are a limited number of economic evaluations of worksite mental health interventions available [28]. The afore-mentioned RCTs on web-based interventions for managing stress [13-16,18,21-24] have not included full economic evaluations.

A number of studies of web-based stress-management interventions have been undertaken; however, little is known about moderators of treatment outcome. It is essential to know for which people with which personal characteristics, which training is most effective under what circumstances in order to optimize training circumstances for each individual based on his or her personal characteristics. Therefore, we include a variety of measurements that are assumed to be related to stress, such as emotion regulation skills [86,87], sleep disturbances [6], psychological detachment or worrying [62,88,89].

### Strengths

A major strength of the current study is the robust randomized controlled trial design, which is the gold standard for clinical trials. Furthermore, this is the first study in Germany to examine the efficacy and cost-effectiveness of a web-based stress-management intervention. We also include a large number of participants in this study to ensure sufficient power.

### Limitations and future directions

One limitation of this study is that no objective measurement of stress (e.g., cortisol levels) will be included. Due to feasibility limitations, only self-report measurements will be examined. Although self-reports always carry the risk of introducing subjective biases, it has been suggested that replacing self-reports with stress-related physiological measurements is not promising [90]. However, the completion of self-reports can be complemented with other measurements to provide more reliable data [90].

Another limitation of the proposed study is the potential self-selection bias. Participants will be eligible to apply for the intervention if they experience work-related stress. One must assume that the individuals who participate in this study possess certain characteristics. Therefore, the results of the current trial will only be applicable to persons who select themselves for this stress-management training.

For future studies it is worthwhile to investigate how much support is needed within the training to achieve the optimal trade-off between treatment outcome and economical costs, i.e., to maximize treatment outcome and simultaneously maintain the lowest level of support possible. A meta-analysis showed that interventions without an eCoach can be effective; however, the effect sizes without an eCoach are much smaller than those of interventions with an eCoach [12]. The current training includes a personal feedback from an eCoach after each of the seven sessions. It would be interesting to examine the size of the treatment effects that result from the application of other, less intensive and more economical support concepts, such as feedback on demand.

In the current trial, we will examine the efficacy of a guided self-help training in comparison to a waitlist control group. This design is therefore not suited to draw any conclusions regarding the effectiveness of the training compared to a face-to-face training, as has been examined elsewhere [27]. In a systematic review and meta-analysis, guided self-help programs for depression and anxiety have been found to have effects similar to those of face-to-face therapies [91]; thus, an investigation of the efficacy of a face-to-face version of the training compared to the web-based training would be worthwhile.

## Conclusions

This study aims to evaluate the efficacy and cost-effectiveness of a newly developed stress-management training for employees. If successful, this training could be made available to a large number of employees because of the low threshold accessibility and potentially low costs.

### Ethical considerations

This study has been approved by the ethics committee of the Philipps University Marburg (registration number AZ-2012-43K).

## Abbreviations

RCT: Randomized controlled trial; PSS-10: Perceived stress scale; DRKS: Deutsches Register für Klinische Studien; TAU: Treatment as usual; ART: Affect regulation training; CES-D: Center for Epidemiological Studies Depression Scale; HADS: Hospital Anxiety and Depression Scales; MBI-GS-D: Maslach Burnout Inventory; ERSQ-27: Emotion regulation skills questionnaire; ERSQ-ES: Emotion regulation skills questionnaire - emotion specific version; ATSPPHS-SF: Attitudes toward seeking professional psychological help scale – short version; (UWES): Utrecht work engagement scale; REQ: Recovery experience questionnaire; ISI: Insomnia severity index; PSWQ-PW: Penn state worry questionnaire-past week; QUALYs: Quality-adjusted life years; EQ-5D: EuroQol; SF-12: Short form 12; TiC-P: Trimbos and institute of medical technology assessment cost questionnaire for psychiatry; ERI-SF: Effort reward imbalance questionnaire – short form; PATHEV: Patient questionnaire on therapy expectation and evaluation; CSQ-8: Client satisfactory questionnaire; CONSORT: the Consolidated standards of reporting trials; NNT: Number needed to treat; ICER: Incremental cost-effectiveness ratio

## Competing interests

Professor Berking is minority shareholder of Minddistrict GmbH which will provide the online training platform.

## Authors’ contributions

MB obtained funding for this study. All authors contributed to the design of the study. EH, DL, DE and SN developed the intervention content. EH wrote the draft of the manuscript. HR supervised the writing process. All authors contributed to the further writing of the manuscript and approved the final version of the manuscript.

## Pre-publication history

The pre-publication history for this paper can be accessed here:

http://www.biomedcentral.com/1471-2458/13/655/prepub

## Current status

Recruitment began on March 1, 2013 and will continue through October 2013. Trial findings at post-test are likely to be available in November 2013.
